# Effect of Digital Tools on the Knowledge and Performance of Frontline Health Workers For Diabetes Control in Myanmar: Cost-Effective Analysis and Quasi Experimental Study

**DOI:** 10.2196/72230

**Published:** 2025-06-16

**Authors:** Kyi Thar, Sathirakorn Pongpanich, Min Nwe Tun

**Affiliations:** 1 College of Public Health Sciences, Chulalongkorn University, Sabbasastravicaya Building (10th - 12th Floor), Soi Chulalongkorn 62, Phyathai Rd, Pathumwan, Bangkok, 10330, Thailand, 66 2 218-8194; 2 Diabetes Foundation Myanmar, Yangon, Myanmar

**Keywords:** diabetes mellitus, digital tools, cost-effectiveness analysis, health personnel, Myanmar

## Abstract

**Background:**

Diabetes has become a significant global health issue, particularly imposing a deep economic burden on low-income countries. Innovative and integrated digital solutions can reduce the impact of diabetes and enhance the quality of care. However, digital solutions have not been utilized before in Myanmar.

**Objective:**

This study aimed to demonstrate the novel integrated effect of diabetes knowledge and registry tools on the performance of front-line health workers in primary health care settings.

**Methods:**

A quasi-experimental study with an intervention and a control group was conducted in two townships from October 2022 to April 2023. For the first time, researchers trained the intervention group to use digital tools for diabetes control and performed monthly follow-ups. The study employed multiple linear regression models to explore the novel impact of digital tools on knowledge and performance scores, their correlations, and their association with covariates. Additionally, it assessed the cost-effectiveness of the intervention by using self-administered questionnaires as measurement tools formulated based on the National Diabetes Guidelines.

**Results:**

A total of 96 participants were enrolled in the study, divided evenly into the two groups. The intervention group exhibited a significant increase in the mean knowledge scores from 85.81 to 99.25 (*P*<.001) and performance scores from 71.22 to 107.16 (*P*<.001). The intervention accounted for 43.2% of the variance in knowledge scores and 62.5% in performance scores (*P*<.001). A positive correlation was found between knowledge and performance scores (*r*=0.45, *P*<.001). The intervention was also cost-effective, with a cost-effectiveness analysis value of 0.711 and an incremental cost-effectiveness ratio of 10127.04 Kyats (US$ 4.83).

**Conclusions:**

As the new integrated intervention yields significant economic gains and positive effects, researchers suggest policy makers replicate this intervention as a nationwide program and recommend scaling up the use of digital tools to improve knowledge and performance for diabetes control in frontline health workers.

## Introduction

### Global Situation Regarding Diabetes

Diabetes is a life-threatening chronic disease that requires effective and sustainable care and treatment. In 2021, it was responsible for 6.7 million deaths worldwide, and the number of people affected was estimated at 537 million, projected to rise to 783 million by 2045 [1]. Despite the increasing burden of diabetes, there is a shortfall of 5.9 million health care professionals required to provide quality care for people living with diabetes [2]. Moreover, many low-income countries face challenges in translating evidence-based knowledge, cost-effective guidelines, and electronic records into actionable solutions to enhance the ability of frontline health workers to deliver quality diabetes services [3].

Health IT has great potential for enhancing diabetes management by saving time and costs involved in data interpretation. Digital knowledge tools can serve as an effective resource for bridging knowledge gaps among health care providers; however, the integrated effect of knowledge tools and diabetes registry on provider performance is not well known [4]. The World Health Organization (WHO) indicates that only 50% of countries use electronic diabetes registries and expects national data to be standardized when registry coverage exceeds 75% [5]. Many low-income countries still struggle to provide comprehensive digital tools for knowledge and registries for frontline health workers. Myanmar should seize these opportunities to improve community-level diabetes care management.

Cost-effective interventions are urgently needed to address the diabetes burden, which cost US$ 966 billion globally in 2021. Southeast Asia’s expenditure was significantly lower at US$ 10.1 billion, compared to North America’s US$ 414.5 billion [6]. Therefore, it is vital to implement high-impact and affordable solutions in low-income countries. A meta-analysis shows that digital tools for diabetes knowledge can be cost-effective [7], yet there is limited evidence in low-income settings [8]. Therefore, it is essential to perform integrated and interdisciplinary research on digital tools for the effective implementation of diabetes control programs in the region.

### Myanmar Context for Diabetes

Myanmar is one of the countries in Southeast Asia with a high burden of diabetes, with a prevalence among 10.5% of the population, which is comparatively higher than that in other countries in the region [9]. A diabetes prevalence survey conducted in 2014 revealed that the burden of the disease had doubled over a decade, and there were no effective strategies or guidelines implemented to raise awareness about diabetes management [10]. Additionally, health workers need to enhance their knowledge, and further research is necessary to improve the quality of diabetes control services at the primary health care level [11].

The rapid growth of mobile technology in Myanmar has created new opportunities for digital health. In 2014, the Ministry of Health established an electronic health management information system and a real-time District Health Information System for all townships. They also distributed 26,000 tablets with essential guidelines for frontline health workers [12]. However, the digital health information system is still in its early stages, and no specific digital application for the diabetes control program exists.

### Objectives

Unlike other studies, this study aimed to evaluate the novel integrated effects of digital tools on diabetes knowledge and registry in relation to diabetes control performance among frontline health care workers in Myanmar. The primary outcomes of the study were the knowledge and performance levels of the health workers, while the secondary outcome was the cost-effectiveness of the intervention.

### Conceptual Frameworks

Researchers designed the study based on two main theories: attribution theory, which examines how knowledge affects diabetes management [13]; and an economic principle assessing the cost-effectiveness of digital health technology [14]. The research hypothesized that integrated digital tools would enhance frontline health workers’ knowledge and performance by reducing diabetes program costs.

## Methods

### Study Design

A quasi-experimental study was conducted in Naypyitaw, the capital of Myanmar, between October 2022 and April 2023 (spanning 6 months). Two townships were selected for the intervention and control groups based on matched population characteristics, geographical conditions, and access to essential diabetes control packages offered by the diabetes control program. The selection criteria for the study areas included a high unknown prevalence of diabetes among the population and inadequate knowledge among health workers [15]. Baseline and endline assessments were carried out for both the groups.

### Participants

Frontline health staff, including midwives, lady health visitors, and public health supervisors, were selected for the study based on specific inclusion criteria: involvement in the diabetes control program, ability to use digital tools, and willingness to participate. Exclusion criteria included those absent for over 1 month, nearing retirement, deemed unfit for intervention, or not approved by supervisors. Using G*Power software (version 3.1.9.2; Heinrich-Heine-Universitat Dusseldorf), the sample size was calculated for multiple linear regression with a 95% CI and power. The reference minimum effect size of the intervention on diabetes control was *F*_4,75_=.25 [16]. The minimum sample required was 86 participants, and 96 were recruited to account for dropouts, finally assigning 48 participants to each group.

### Interventions

The new intervention involved integrating the two new digital tools developed by the authors, Myanmar Diabetes Guides and Digital Registry. This is unique and significant because other studies measure the silo effects [17]. Researchers installed digital tools and provided 3 days of intensive training on how to use the tools. Additionally, researchers conducted monthly follow-ups and provided reorientation sessions to the intervention group.

#### Myanmar Diabetes Guide

This is a new comprehensive bilingual knowledge tool developed by researchers in collaboration with the National Diabetes Control Program. According to the WHO and National program guidelines, the tool addresses the risks and promotes the health, screening, diagnosis, care and treatment, and complication referrals. It includes interactive patient dialogues, video demonstrations, and diabetes-related wikis. The tool is open to access in both online and offline settings and can be used on mobile tablets [18].

#### Diabetes Registry Tool

The electronic diabetes registry tool was designed using Kobo Toolbox, an open-source tool for field data collection in humanitarian response [19]. This tool collects vital patient information for diabetes management programs and replaces paper-based reporting. The tools enable health workers to trace risk factors, analyze data, calculate prevalence, identify complications, and estimate the requirement of diabetes-related commodities. The application is available for online and offline use and is compatible with both tablets and computers with a user password to protect data privacy [19].

#### Training for Utilization of the Application

Researchers conducted training on the orientation for using the digital tools that consisted of three components with lectures, demonstration, and practice sessions on (i) health promotion, identifying high-risk individuals, and establishing volunteer networks; (ii) training for the diabetes knowledge tools; and (iii) training for the diabetes registry tool.

### Measurement Instruments

The researchers developed self-administered questionnaires for data collection. The questionnaire was created in English and subsequently translated into the Burmese language. It included a scoring system for the primary outcomes, which assessed the knowledge and performance in five key domains of diabetes management: (i) health promotion, (ii) diabetes screening, (iii) care and treatment, (iv) referral, and (v) reporting, all referencing the WHO and National Diabetes Guidelines. The researchers established an expert panel to review the questionnaire to ensure content validity. This panel included a diabetes program manager, an expert clinician, and township health officers. The validity index for the questionnaire was scored at 0.6. Additionally, the reliability of the questionnaire was pretested by two different observers, with an interrater kappa value of 0.68 between the two observers.

The WHO-CHOICE (WHO’s Choosing Interventions that are Cost-Effective) Analysis tool for noncommunicable diseases was used to measure costing data [20]. This costing data included direct costs (intervention costs, program costs, and treatment costs) and indirect costs (communication, consulting, value of time, and work). The cost-effectiveness analysis aimed to demonstrate the economic benefits of the intervention for future investments.

### Data Collection and Statistical Analysis

The principal investigator and two research assistants collected baseline and endline data. Before data collection began, the study’s purpose was explained to authorities and participants. Primary cost data were obtained from participants, while secondary data on treatment costs came from the township hospital and program costs from the township health departments. The team checked the accuracy of the questionnaires, addressed any missing responses, and cross-verified participant data with the secondary data. Health assistants from the townships were followed-up monthly on using the tools.

Data analysis was conducted using SPSS software (version 22.0; IBM Corp). Knowledge and performance scores followed the National Diabetes Control Guideline criteria. Sociodemographic data differences were assessed with the χ^2^ test, mean outcome data with the *t* test, and outcome correlations with the Pearson correlation test. The impact of the intervention was evaluated using multiple logistic regression analysis. All tests were statistically significant at a 95% CI. Cost-effectiveness was assessed through the cost-effectiveness ratio and incremental cost-effectiveness ratio.

### Ethical Considerations

The study received approval from the Chulalongkorn University Ethics Review Committee (090.2/64, COA No. 177/2022). The Ministry of Health, Naypyitaw Department of Health, granted permission for data collection (NPT/NCD/007-2021/5925). Participation in the research was voluntary; informed consent was obtained, and data confidentiality was ensured. During the registration, an internet package (US$ 2) and a 50 pcs box of surgical masks were provided to participants as compansation for participating the research.

## Results

### Patient Inclusion

**Figure 1.**
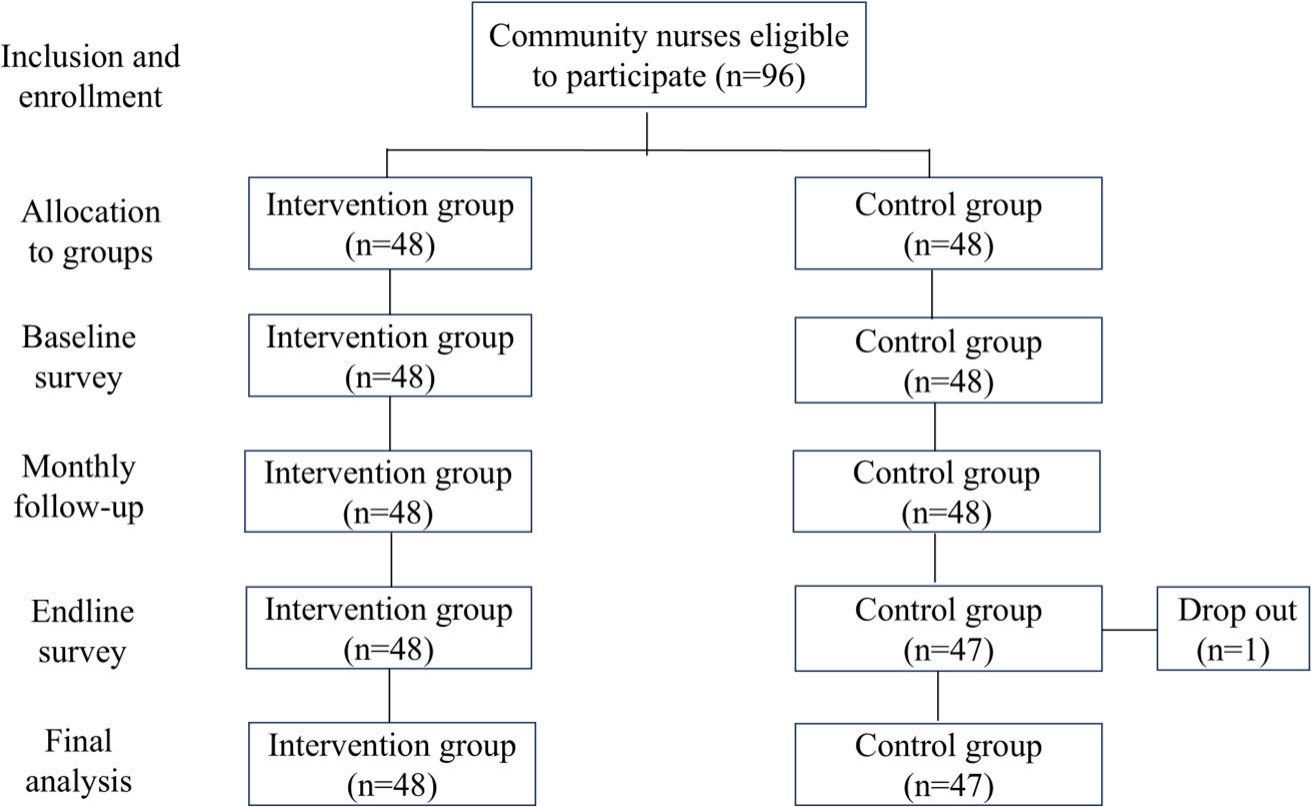
Flow chart for quasi-experimental design.

Ninety-six participants enrolled in the study, and 1 patient dropped out. Figure 1 shows the flow chart of the quasi-experimental study design.

### Participant Sociodemographic Characteristics

The study used the *χ*^2^ test to examine participant characteristics in relation to the sociodemographic variables, work-related variables, and variables related to diabetes control (Table 1). Significant differences between the two groups were observed based on gender (*P*=.02), job designation (*P*=.02), distance from the township (*P*=.001), and duration of internet use (*P*=.001). Independent *t* tests analyzed differences in mean knowledge and performance scores. The mean knowledge scores were statistically associated with gender (*P*=.001), job designation (*P*<.001), and diabetes control training (*P*=.02). The mean performance scores were statistically associated with gender (*P*=.04). job designation (*P*=.009), diabetes control training (*P*=.02), diabetes registry training (*P*=.02), number of postings (*P*=.03), level of facilities (*P*=.02), and experiences with diabetes campaigns (*P*=.007).

**Table 1.** Sociodemographic variables of the participants (n=96).

Sociodemographic variables	Total (n=96)	Intervention group (n=48)	Control group (n=48)	*P* value
Age (years), mean (SD)	32.71 (9.52)	31.75 (9.18)	33.67 (9.55)	.33^a^
Gender, n (%)				.02^b^^,^^c^
Male	14 (14)	3 (6.3)	11 (22.9)	
Female	82 (82)	45 (93.7)	37 (77.1)	
Marital status, n (%)				.33^b^
Single	31 (32.3)	14 (29.2)	17 (35.4)	
Married	65 (67.7)	34 (70.8)	31 (64.6)	
Educational status, n (%)				.58^b^
High school	24 (25)	11 (22.9)	13 (27.1)	
Graduate	72 (75)	37 (77.1)	35 (72.9)	
Designations, n (%)				.02^b^^,c^
Lady health visitors	8 (8.3)	4 (8.3)	4 (8.3)	
Midwives	55 (57.3)	34 (70.9)	21(43.8)	
Public health supervisor	33 (34.4)	10 (20.8)	23 (47.9)	
Number of postings, n (%)				.73^b^
1st posting	55 (57.3)	27 (56.3)	28 (58.3)	
2nd-5th posting	30 (31.3)	16 (33.3)	14 (29.2)	
>5th posting	11 (11.4)	5 (10.4)	6 (12.5)	
Level of facilities, n (%)				.74^b^
Township	19 (19.8)	11 (22.9)	8 (16.7)	
Rural health center	21 (21.9)	10 (20.8)	11 (22.9)	
Subcenter	56 (58.3)	27 (56.3)	29 (60.4)	
Distance from township, n (%)				.001^b^^,^^c^
≤10 miles	55 (57.3)	19 (39.6)	36 (75)	
>10 miles	41 (42.7)	29 (60.4)	12 (25)	
DM^d^ control training, n (%)				.50^b^
Received before	67 (69.8)	33 (68.8)	34 (70.8)	
Never received	29 (30.2)	15 (31.2)	14 (29.2)	
DM registry training, n (%)				.21^b^
Received before	47 (49)	26 (54.2)	21 (43.8)	
Never received	49 (51)	22 (45.8)	27 (56.2)	
DM campaign experiences, n (%)				.11^b^
Received before	57 (59.4)	25 (52.1)	32 (66.7)	
Never received	39 (40.6)	23 (47.9)	16 (33.3)	
Duration of internet usage, n (%)				.001^b^^,^^c^
≤5 years	49 (51)	35 (72.9)	14 (29.2)	
>5years	47 (49)	13 (27.1)	34 (70.8)	

a independent t-test.

b *χ*2 test.

c statistically significant *P*<.05.

d DM: diabetes mellitus.

### Effect of Intervention on Knowledge

**Figure 2.**
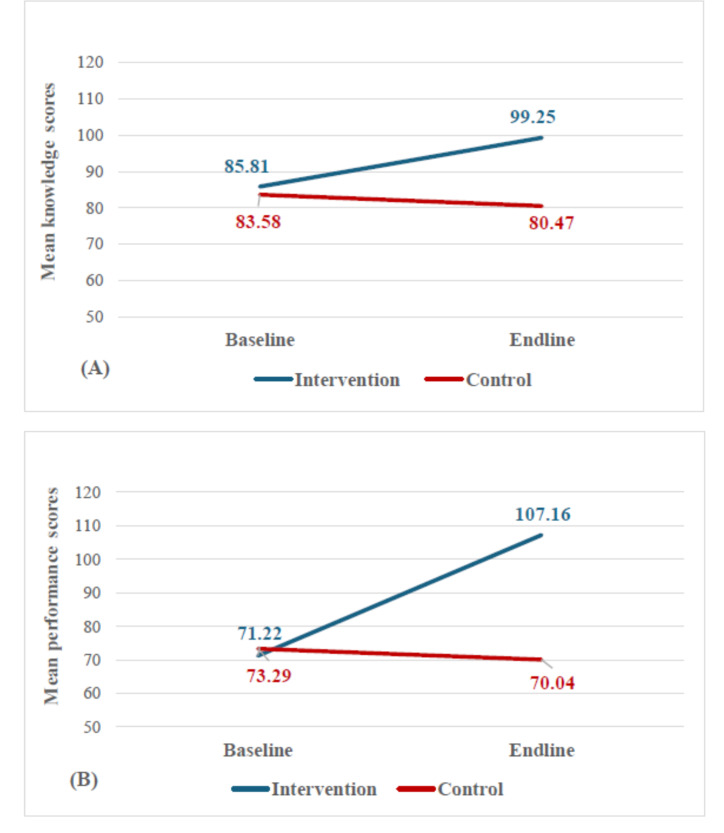
Comparison of mean outcomes between the intervention and control: (A) mean knowledge scoring, (B) mean performance scoring.

An average of 94.2% of the intervention group (45/48) regularly utilized diabetes knowledge tools. During the baseline assessment, no significant difference in the mean knowledge scores was noted between the two groups (*P*=.20). However, a significant difference emerged between the two groups at the endline (*P*<.001). The mean (SD) knowledge score in the intervention group rose significantly from 85.04 (9.73) to 99.25 (5.33; *P*<.001). In contrast, the mean (SD) knowledge score in the control group slightly declined from 83.58 (11.17) to 80.47 (16.99) (Figure 2); however, this change was not significant (*P*=.21; Table 2). The multiple linear regression model, after adjusting for potential confounding factors, showed a significant effect, with an adjusted *r*² of 0.43, an unstandardized β coefficient of 17.769, and a standardized β coefficient of 0.569 (*P*<.001; Table 3).

**Table 2.** Comparison of mean outcome variables between the intervention and control groups.

Outcome variable	Total Mean (SD) N ^Ba^=96 N ^Eb^=95	Intervention group Mean (SD) N ^B^=48 N ^E^=48	Control group Mean (SD) N ^B^=48 N ^E^=47	*P* value^c^
Knowledge Scoring				
Total knowledge scoring ^B^	85.04 (9.73)	85.81 (8.20)	83.58 (11.17)	.20
Total knowledge scoring ^E^	87.25 (21.67)	99.25 (5.33)	80.47 (16.99)	<.001^d^
Health promotion ^B^	20.01 (2.51)	20.00 (2.37)	19.95 (2.71)	.76
Health promotion ^E^	20.22 (4.83)	22.35 (1.53)	19.31 (3.96)	<.001^d^
Screening and diagnosis ^B^	19.23 (3.52)	19.25 (3.55)	19 (3.44)	.55
Screening and diagnosis ^E^	19.93 (5.34)	22.68 (2.41)	18.41 (4.46)	<.001^d^
Care and treatment ^B^	16.82 (3.12)	17.68 (2.69)	15.97 (3.29)	.08
Care and treatment ^E^	18.15 (5.46)	21.54 (1.67)	15.72 (4.55)	<.001^d^
Referral of severe cases ^B^	14.34 (2.44)	14.43 (1.85)	14.10 (2.91)	.47
Referral of severe cases ^E^	14.63 (3.94)	16.46 (1.43)	13.50 (3.42)	<.001^d^
Reporting and registry ^B^	14.52 (2.24)	14.43 (2.21)	14.54 (2.33)	.89
Reporting and registry ^E^	14.45 (3.86)	16.21 (1.50)	13.52 (3.19)	<.001^d^
Performance scoring				
Total performance scoring ^B^	72.33 (9.73)	71.22 (28.35)	73.29 (35.66)	.75
Total performance scoring ^E^	88.50 (33.50)	107.16 (15.62)	70.04 (29.72)	<.001^d^
Health promotion ^B^	9.23 (4.22)	9.54 (3.99)	8.91 (4.23)	.46
Health promotion ^E^	10.59 (3.8)	12.42 (2.15)	8.77 (3.44)	<.001 ^d^
Screening and diagnosis ^B^	9.41 (4.32)	9.38 (4.28)	9.42 (4.26)	.97
Screening and diagnosis ^E^	10.37 (4.1)	12.56 (2.89)	8.17 (3.13)	<.001^d^
Care and treatment ^B^	38.93 (20.83)	37.17 (19.57)	40.54 (22.06)	.43
Care and treatment ^E^	49.01(21.6)	59.08 (12.21)	38.95 (21.17)	<.001 ^d^
Referral of severe cases ^B^	4.24 (4.14)	3.89 (4.21)	4.73 (4.21)	.34
Referral of severe cases ^E^	5.37 (5.3)	7.13 (6.24)	3.60 (3.49)	.001^d^
Reporting and registry ^B^	10.42 (6.23)	11.23 (5.68)	9.56 (6.58)	.19
Reporting and registry ^E^	13.24 (6.5)	15.95 (4.20)	10.54 (6.78)	<.001^d^

a B=baseline.

b E=endline.

c independent t-test.

d statistically significant *P*<.05.

**Table 3.** Effect of intervention on total knowledge scoring after adjusting for all possible confounding variables (full model multiple linear regression analysis).

Variables	Total knowledge score at endline (n=95)					
	β	SE	95% CI	Standardized β coefficient	*t* test *(df)*	*P* value
Intervention township	17.767	2.942	11.92 to 23.61	0.569	6.040 (7)	<.001^a^
Baseline knowledge score	.497	0.137	0.23 to 0.77	0.311	3.630 (7)	<.001^a^
Gender	−1.876	3.932	−9.69 to 5.93	−0.042	−0.477 (7)	.63
Designation of work	2.726	2.168	−2.69 to 7.96	0.105	1.258 (7)	.21
Distance from township	2.633	2.697	−3.50 to 7.29	0.083	0.983 (7)	.33
Duration of internet usage	.042	0.423	−0.79 to 0.88	0.009	0.100 (7)	.92
Diabetes control training	6.004	2.745	0.549 to 11.46	0.177	2.187 (7)	.03^a^

a significant at *P*<.05.

b r2=0.472.

c Adjusted r2=0.430.

d Fitness Sample Corrected Akaike’s Information Criterion (AICC)=758.765.

### Effect of Intervention on Performance

A total of 91.2% (44/48) of the intervention group regularly used the registry tool and registered 1747 diabetes patients within 6 months. The mean (SD) performance score in the intervention group significantly increased from 71.22 (28.35) to 107.16 (15.62; *P*<.001). In contrast, the control group experienced a decrease in the mean (SD) performance scores from 73.29 (35.66) to 70.04 (29.72; *P*=.22). At baseline, the two groups had no significant difference in the mean performance scores (*P*=.75). At the endline, a significant difference was noted between the two groups (*P*<.001; Table 1). A multiple linear regression model, adjusted for potential confounding factors, indicated a significant effect, with an adjusted *r*² of 0.642, an unstandardized β coefficient of 33.143, and a standardized β coefficient of 0.554 (*P*<.001; Table 4).

**Table 4.** Effect of intervention on the total performance score after adjusting for all possible confounding variables (full model multiple linear regression analysis).

Variables	Total performance score at endline (n=95)					
	β	SE	95% CI	Standardized β coefficient	*t* test *(df)*	*P* value
Intervention township	33.143	4.520	24.15 to 42.13	0.554	7.332 (11)	<.001^a^
Baseline performance score	.499	0.67	0.36 to ‐0.63	0.532	7.483 (11)	<.001^a^
Gender	8.706	6.134	−3.49 to 20.90	0.103	1.419 (11)	.16
Designation of work	3.054	4.103	−5.11 to 11.21	0.061	0.744 (11)	.46
Distance from township	7.419	4.247	−1.03 to 15.86	0.123	1.747 (11)	.08
Duration of internet usage	−.670	0.668	−1.99 to 0.66	−0.71	−1.003 (11)	.32
Diabetes control training	−.339	5.795	−11.86 to 11.18	−0.005	−0.058 (11)	.95
Diabetes registry training	1.886	5.210	−8.47 to 12.25	0.31	0.362 (11)	.72
Diabetes campaign experience	−.170	4.319	−8.76 to 8.42	−0.003	−0.039 (11)	.97
Level of facility	2.906	2.552	−2.17 to 7.98	0.077	1.138 (11)	.26
Number of postings	−.773	2.210	−5.167 to 3.62	−0.028	−0.350 (11)	.73

a significant at *P*<.05.

b *r*2=0.684.

c Adjusted *r*2=0.642.

d Fitness Sample Corrected Akaike’s Information Criterion (AICC)= 845.994.

### Correlation Between Knowledge and Performance

**Figure 3.**
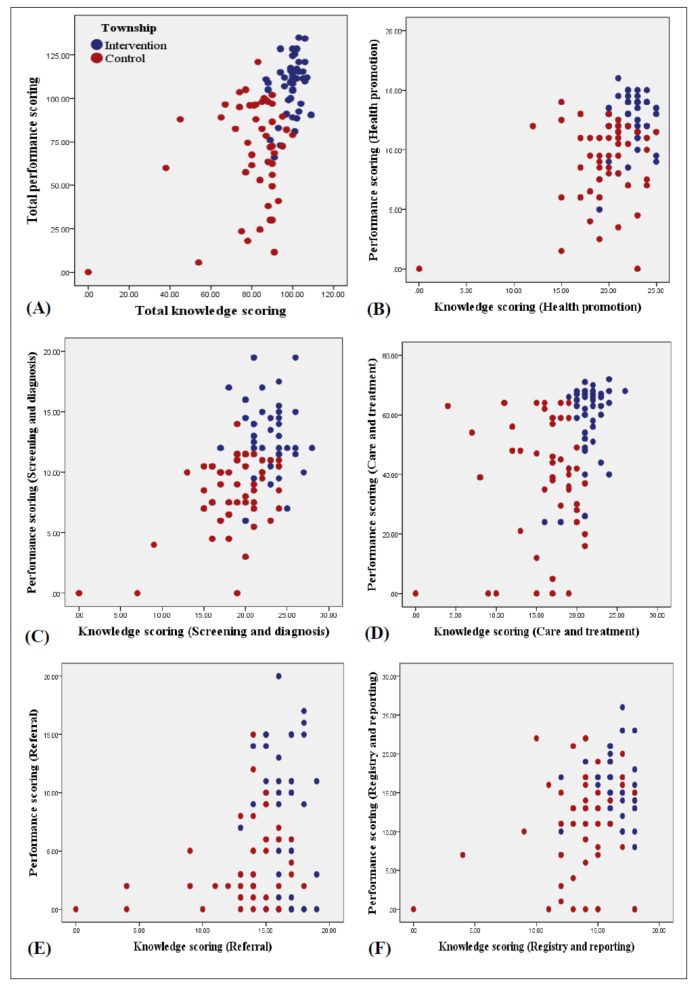
Correlation between mean knowledge and performance scoring (n=95). (A) Total scoring, (B) health promotion, (C) screening and diagnosis, (D) care and treatment, (E) referral, (F) registry and reporting.

The study showed a significant positive correlation between total knowledge and performance scores, with a correlation coefficient of *r*=0.45 (*P*<.001) at the endline (Figure 3). A significant correlation was found in the intervention group compared to the control group, with *r*=0.34 (*P*=.02). However, no significant correlation was observed in the control group, with *r*=0.02 (*P*=.89)

### Cost-Effectiveness of the Intervention

**Figure 4.**
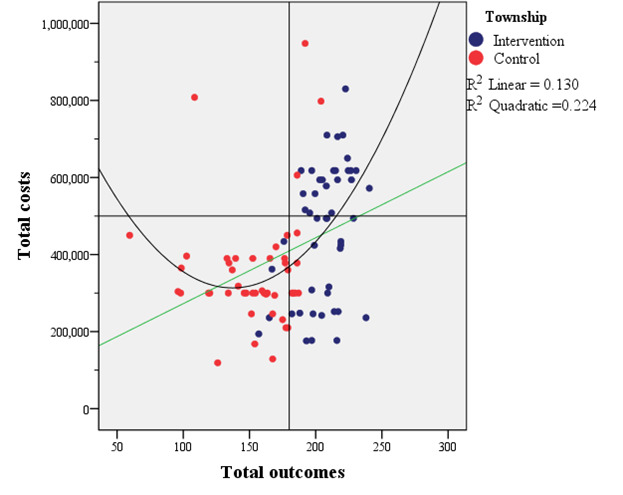
Cost-effectiveness between the intervention and control.

Researchers categorized the cost data into three main categories: (1) intervention costs, which included web application development, training, internet usage, and stationery; (2) program implementation costs, covering travel expenses, costs for consultation and communication, loss of income due to diabetes-related work, and other miscellaneous costs; and (3) treatment costs for complications. The total cost for the intervention group was 22,213,000 Kyats (US$ 10,586.71), while the control group incurred a total cost of 22,779,000 Kyats (US$ 10,856,4714) (Table 5). The intervention was deemed cost-effective, with a cost-effectiveness ratio of 0.711, and it was considered cost-effective when the cost-effectiveness ratio was less than 1. The study also analyzed the incremental cost-effectiveness ratio for comparative investment. The incremental cost-effectiveness ratio indicated saving 10,127.04 Kyats (US$ 4.83) for both outcomes, 30,154.50 Kyats (US$ 14.40) for knowledge, and 15,247.84 Kyats (US$ 7.27) in performance. The visibility of cost-effectiveness was demonstrated by plotting bootstrap results against outcomes and expenses (Figure 4).

**Table 5.** Summary of costing and outcome for cost-effective analysis.

Overall and detailed costs	Intervention group	Control group
Intervention cost (Kyats)^a^		
Training and software cost	2,000,000 (US$ 953.20)	0 (US$ 0)
Internet cost	4,662,000 (US$ 221.91)	1,878,000 (US$ 895.05)
Stationary cost	949,000 (US$ 452.29)	1,262,000 (US$ 601.47)
Sub total	7,611,000 (US$ 3627.40)	3,140,000 (US$ 1496.52)
Hospital expense (Kyats)		
Care and treatment cost due to diabetes complications	4,600,000 (US$ 2192.36)	6,000,000 (US$ 2859.6)
Sub total	4,600,000 (US$ 2192.36)	6,000,000 (US$ 2859.6)
Staff expense (Kyats)		
Travel cost	2,460,000 (US$ 1172.44)	3,484,000 (US$ 1660.47)
Consultation cost for diabetes	1,825,000 (US$ 869.79)	2,524,000 (US$ 1202.94)
Communication cost	1,716,000 (US$ 817.85)	1,891,000 (US$ 901.25)
Loss of income due to extra workload	1,280,000 (US$ 610.05)	2,807,000 (US$ 1337.82)
Miscellaneous cost	2,721,000 (US$ 1296.83)	2,933,000 (US$ 1397.87)
Sub total	10,002,000 (US$ 4766.95)	13,639,000 (US$ 6500.35)
Total cost (Kyats)	22,213,000 (US$ 10,586.72)	22,779,000 (US$ 10,856.47)
Outcomes		
Outcome scoring		
Mean knowledge scoring	99.25	80.48
Mean performance scoring	107.16	70.04
Total outcome score	206.41	150.52

a A currency exchange rate of Kyat 1=US $0.72 is applicable.

## Discussion

### Principal Findings

The intervention of new integrated digital tools saved costs and yielded significant positive outcomes in knowledge and performance of diabetes control among frontline health workers. Although the study is quasi-experimental, the researcher minimized confounding by matching selection criteria and adjusting covariates through multiple linear logistic regression. Consequently, the study achieved its goals through a consistent design and reliable analytical methods, resulting in valid outcomes. The overall results align with other research on digital solutions to enhance diabetes control [21].

### Comparison With Previous Studies

#### Digital Knowledge Solution for Diabetes Control

This study significantly observed that using integrated digital tools could enhance five key domains of knowledge and performance related to health promotion, screening, care and treatment, referral, and reporting diabetes in primary health care settings. Integrating digital knowledge tools and a registry tool is an effective intervention for diabetes control among frontline health workers. A systematic review of evidence-based medicine found that digital knowledge tools can improve diabetes control knowledge among primary health care staff [22]. Digital tools can improve the screening process, as supported by other meta-analyses regarding the performance of diabetes screening [23]. Additionally, positive effects of digital tools on providers’ performance were observed in areas such as reminders, clinical care decisions, glycemic control, and web-based training and education programs [24].

While this study concentrated solely on the provider side, digital tools can offer numerous patient benefits, such as increased awareness, improved understanding, and enhanced self-management skills within the community [25]. However, a meta-analysis conducted in Southeast Asia found that patients’ knowledge of diabetes was inadequate, especially among women with low education levels and poor diabetes control [26]. Therefore, further research and tailored training in digital interventions are recommended to improve knowledge and awareness among patients, their families, and the community.

#### Diabetes Registry for Electronic Health Records

A meta-analysis using electronic health records for diabetes across 12 countries showed positive outcomes [27]. A diabetes registry can enhance the quality of patient care in rural areas, both in high-income countries like the United States of America [28] and in various low-income countries [29]. Unlike other studies, this research connected the positive results of using a digital diabetes registry and knowledge tools in routine diabetes program reporting, especially for resource-limited settings. The findings showed that reporting performance was significantly improved, and several patients were registered correctly. Therefore, the authors recommend adopting an open-resource, low-cost digital diabetes registry as a nationwide program for diabetes control in Myanmar and other low-income countries.

#### Cost-Effectiveness on Diabetes Interventions

This study is significant because it measures the direct and indirect costs associated with diabetes management using the WHO-CHOICE formula. Furthermore, the cost analysis was conducted on both knowledge and performance outcomes. Digital monitoring for diabetes has gained popularity alongside increased access to high-speed internet. This advancement has helped reduce costs, lower the number of hospital visits, save time, and improve the quality of life for those managing diabetes [30]. However, some studies suggest that the cost categories related to diabetes are too complex to provide accurate data [31,32]. Additionally, other research indicates that cost analyses may be inadequate due to factors, such as underlying socioeconomic conditions, underreporting, the severity of complications, and the long-term effects of diabetes [32].

Generally, an intervention is considered cost-effective when the cost-effectiveness ratio is less than 1. This study demonstrated cost-effectiveness with a cost-effectiveness ratio of 0.711. Similar evidence supporting cost-effectiveness has been observed in consumer-based solutions, digital tools for blood glucose, and diabetes self-management education in the United Kingdom [33]. This study explored the incremental cost-effectiveness ratio to assess the additional investment needed to enhance knowledge and performance scores for diabetes control. In contrast, another study conducted in Sweden examined incremental cost-effectiveness ratio results related to diabetes control through patients’ quality-adjusted life years [31]. Nevertheless, this study urges policy makers to consider further investments in digital tools, even though a sophisticated cost-effectiveness framework has not yet been developed.

### Limitations

According to this study, despite several benefits, the rollout and sustainability of the digital diabetes registry encountered some limitations. Initially, the studies intended to measure baseline, midterm, and endline assessments. However, the authority approved only two measurements based on the country’s political situation and the expectation of no significant variation in the midterm. Although the initial goal was to collect real-time data, health staff could only upload information monthly due to being overburdened with competing priorities. The study focused on Naypyitaw, which limits generalizability to the entire country, and only has a 6-month duration, so it cannot evaluate the long-term impacts. The study population focused solely on public providers, excluding private providers, patients, and the wider community. Furthermore, the digital tools were not interoperable with the District Health Information System.

### Conclusions

The intervention used a multidisciplinary approach for frontline health personnel at the grassroots level, significantly improving knowledge and performance and reducing program costs. Unlike other studies, this research demonstrated the integrated and correlated effects of digital knowledge and reporting tools. Given these strengths and limitations, researchers recommend that policy makers replicate the intervention nationwide, develop clear standard operating procedures, establish a reporting schedule, and provide an internet data package to enhance the use of digital tools. Furthermore, the diabetes registry operates in isolation, necessitating the creation of an interoperable system to connect with the District Health Information System. Additionally, extensive studies on long-term population research and economic evaluations are essential to evaluate the sustainability of digital tools. We suggest engaging outstanding community nurses as champions to share their best practices of digital applications, and these measures could ensure data quality and sustainability of digital tools to enhance diabetes control in Myanmar and other low-income countries.

## Abbreviations

WHO: World Health Organization
WHO-CHOICE: WHO’s Choosing Interventions that are Cost-Effective
